# CcpA Coordinates Growth/Damage Balance for *Streptococcus pyogenes* Pathogenesis

**DOI:** 10.1038/s41598-018-32558-0

**Published:** 2018-09-24

**Authors:** Elyse Paluscio, Michael E. Watson, Michael G. Caparon

**Affiliations:** 1 Department of Molecular Microbiology, Washington University School of Medicine St Louis, St. Louis, MO 63110-1093 United States; 2 Present Address: Department of Microbiology and Molecular Genetics, University of Pittsburgh School of Medicine, Pittsburgh, PA 15219 United States; 3 Present Address: Department of Pediatrics and Communicable Diseases, University of Michigan Medical School, Ann Arbor, MI 48109-5624 United States

## Abstract

To achieve maximum fitness, pathogens must balance growth with tissue damage, coordinating metabolism and virulence factor expression. In the gram-positive bacterium *Streptococcus pyogenes*, the DNA-binding transcriptional regulator Carbon Catabolite Protein A (CcpA) is a master regulator of both carbon catabolite repression and virulence, suggesting it coordinates growth/damage balance. To examine this, two murine models were used to compare the virulence of a mutant lacking CcpA with a mutant expressing CcpA locked into its high-affinity DNA-binding conformation (CcpA^T307Y^). In models of acute soft tissue infection and of long-term asymptomatic mucosal colonization, both CcpA mutants displayed altered virulence, albeit with distinct growth/damage profiles. Loss of CcpA resulted in a diminished ability to grow in tissue, leading to less damage and early clearance. In contrast, constitutive DNA-binding activity uncoupled the growth/damage relationship, such that high tissue burdens and extended time of carriage were achieved, despite reduced tissue damage. These data demonstrate that growth/damage balance can be actively controlled by the pathogen and implicate CcpA as a master regulator of this relationship. This suggests a model where the topology of the *S. pyogenes* virulence network has evolved to couple carbon source selection with growth/damage balance, which may differentially influence pathogenesis at distinct tissues.

## Introduction

For many bacterial pathogens, optimal fitness represents a fine-tuned balance between growth and the degree to which host tissues are damaged. Achieving this balance is challenged by the dynamic nature of infection, the immune response, and the diverse environments encountered at alternative host tissues. Successful pathogens have evolved versatile transcriptional networks capable of coordinating the expression of genes encoding virulence factors with those for metabolism and growth. For many pathogens, this is accomplished by a mechanism that allows for the preferential metabolism of one carbon source over another, known as carbon catabolite repression (CCR) (reviewed^1–3^). Thus, insight into pathogenesis can be gained from an analysis of how CCR contributes to virulence gene regulation.

For *Streptococcus pyogenes* (group A *Streptococcus*), CCR has been shown to play an important role in virulence^4–7^. This Gram-positive pathogen causes a wide variety of diseases at numerous tissue sites, ranging from mild and self-limiting (impetigo, pharyngitis), to severe and life threatening (cellulitis, necrotizing fasciitis), to serious post-infection sequelae (rheumatic fever, acute glomerulonephritis)^8–11^. In large part, its capacity to infect different tissues is due to its ability to interrogate its environment and rapidly alter its gene expression profile^12–18^. Environmental signals sensed by the regulatory network include osmolarity, temperature, pH, salt, and most importantly, nutrient availability^14,19–21^. For this latter cue, CCR plays a significant role, directing the preferential metabolism of more energetically favorable carbon sources at the expense of less favorable carbon sources, primarily through transcriptional repression of the genes necessary for processing alternative carbon sources^4–7,22^.

In *S. pyogenes* and other low G + C Gram-positive bacteria, a major pathway for CCR involves a DNA-binding transcription factor known as Catabolite Control Protein A (CcpA) (reviewed^23^). Numerous studies have shown that CcpA is a global regulator of gene expression in *S. pyogenes*, affecting transcription of approximately 20% of the total genome, including numerous virulence factors^4–7^. These studies have shown that, while CcpA primarily acts to repress transcription of metabolism and virulence genes, expression of a small subset of the transcriptome, including the well-studied SpeB cysteine protease, is positively regulated by CcpA^4,5^.

The *in vivo* behavior of *S. pyogenes* CcpA mutants has been extensively investigated. In a murine model of soft-tissue infection, the expression of several genes showed distinct expression profiles with respect to time between WT and a mutant lacking CcpA (ΔCcpA)^5^, suggesting that CcpA-mediated modulation of gene expression was essential for optimal fitness. In this model of soft tissue infection, the bacteria grow in a well-defined lesion demarcated by the host’s innate immune response^24^. As a constrained environment, there is likely a limited supply of nutrients, requiring the bacterial community to constantly adjust its metabolism in order to consume less favorable carbon sources as more favored sources are depleted. This suggests that CCR functions as a molecular clock to inform the transcriptional network to balance growth rates with tissue damage in a temporal pattern with respect to carbon source availability. As predicted, the loss of CcpA disrupts temporal patterns of gene expression *in vivo*^4,5^ and results in a significant attenuation of virulence, with decreased levels of tissue damage and bacterial burden in both acute and chronic models of infection^4–7,25^.

A caveat is that these studies were limited to analysis of a *ccpA* null mutant, similar to other studies in *S. pyogenes*^6,7^, other streptococci^26–31^, staphylococci^32,33^, enterococci^34,35^, *Listeria*^36^, *Bacillus anthracis*^37^ and *Clostridium difficile*^38^. As a result we have an incomplete picture of CcpA’s role in pathogenesis. For example, while over-expression of CcpA-repressed genes is associated with attenuation, it is not clear that these genes are required for pathogenesis. Similarly, by linking CCR with virulence, it is hypothesized that fitness is increased by imparting a temporal component to the regulatory network^5^. In this model, CCR links expression of virulence factors required at the initial stages of infection to the metabolism of preferred carbon sources, under the control of CcpA configured into its high-affinity DNA-binding conformation. As infection proceeds, preferred carbon sources are depleted and CcpA switches to its low-affinity conformation to reconfigure metabolism for adaption to alternative carbon sources. Concurrently, CcpA’s low-affinity conformation de-represses expression of virulence genes required for late-stage infection. This model predicts that mutations locking CcpA into its high-affinity conformation characteristic of early-stage infection should also affect pathogenesis, but by a fundamentally different mechanism as compared to null mutations, which more closely mimic the behavior of de-repressed CcpA at later time points.

In the present study, we have analyzed the virulence phenotypes of a CcpA mutant constrained to constitutively adopt its high-affinity DNA-binding conformation independent of any regulatory input to function as a “locked-on” mutant for comparison to a CcpA null mutant, which lacks any CcpA activity. The data strongly support the temporal regulation model, show that mis-regulation can lead to diverse infection outcomes at different tissues, and provide information important for understanding the selective pressures that have configured this network topology.

## Results

### Construction of “locked-on” CcpA

**Figure 1:**
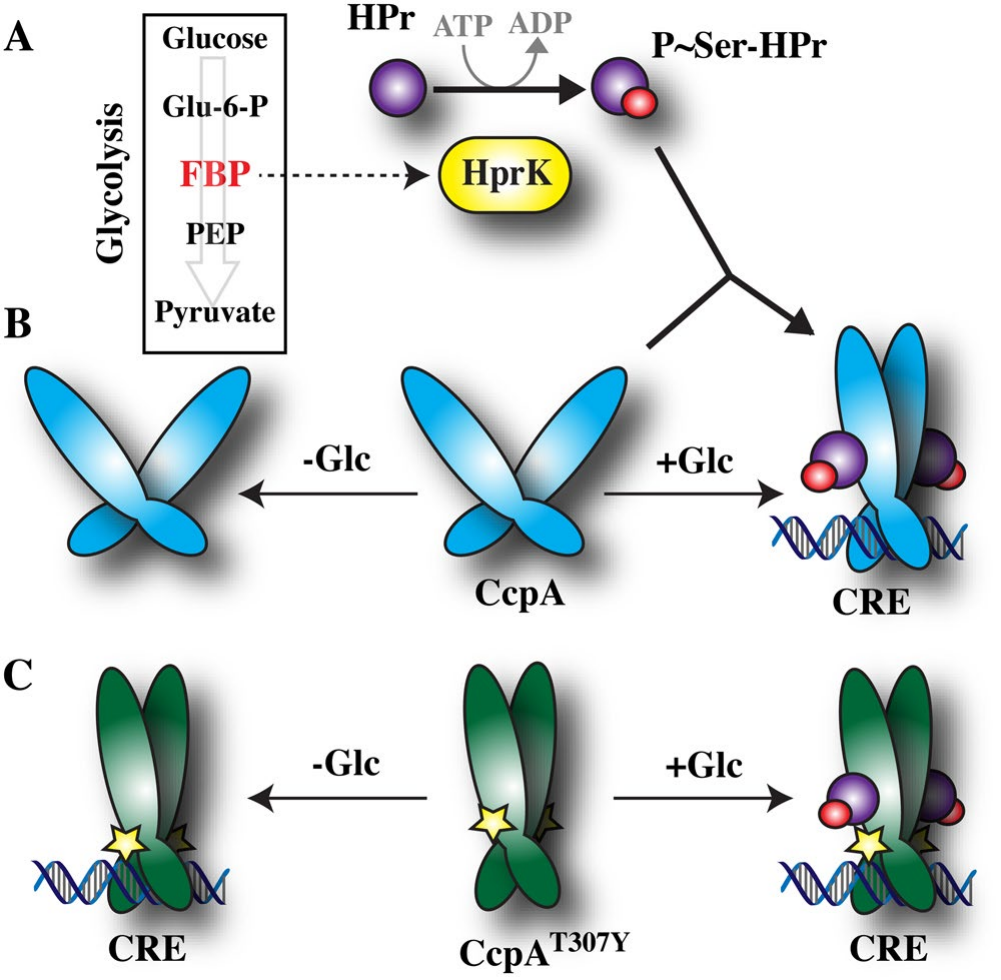
CcpA-mediated CCR and the construction of a “locked-on” mutant. (**A**) As shown in the Figure, metabolism via glycolysis of glucose or other favorable carbohydrates results in elevated levels of the glycolytic intermediate fructose 1,6-bisphosphate (FBP). As an allosteric activator of the enzyme HPr kinase (HPrK), high FPB levels stimulate HPrK to phosphorylate a regulatory residue (Ser_46_) of HPr. (**B**) The resulting product (P~Ser-HPr) binds to a CcpA dimer, inducing a conformational change that reorients the dimer’s DNA-binding domains to adopt a configuration that binds with high-affinity to a conserved DNA sequence known as a catabolite repression element (CRE). Thus, in the presence (+) or absence (−) of glucose (Glc), CcpA has a high or low affinity for binding CRE, respectively. (**C**) Substitution of CcpA Thr^307^ with a Tyr residue (shown by the star symbol) mimics binding of P~Ser-HPr, such that the resulting mutant (CcpA^T307Y^) is constitutively locked into its high-affinity, CRE-binding configuration, irrespective of glucose (+Glc, −Glc) or P~Ser-HPr. Other abbreviations: glucose-6-phosphate, Glu-6-P; phosphoenolpyruvate, PEP.

As a DNA-binding transcription factor, CcpA recognizes a specific semi-palindromic DNA motif known as the catabolite repressor element (CRE) site^23^. In *S. pyogenes*, about two-thirds of the CcpA regulated genes are repressed by CcpA, with the other one-third activated^4–7^. Activation vs. repression is dictated by the location of the CRE site, with the latter located adjacent to the −10 element of the promoter, while for the former, the CRE site is located just upstream of the −35 region of the promoter^23^. The central pathway for carbon metabolism in *S. pyogenes* is the Embden-Meyerhof-Parnas (EMP) glycolytic pathway (Fig. 1A^39^). When a preferred substrate such as glucose is abundant, it is rapidly taken up by the cell and processed, resulting in a high intracellular concentration of the EMP intermediate fructose 1,6-bisphosphate (FBP, Fig. 1A). In turn, high FBP levels act allosterically to influence the enzymatic activity of the enzyme Hpr kinase (HprK, Fig. 1A), which functions both as a kinase and a phosphatase to control the phosphorylation state of the phosphocarrier protein HPr^2,40^. At high FBP levels, the kinase activity of HprK is stimulated to phosphorylate HPr at amino acid residue serine 46 (Ser_46_, Fig. 1A)^2,40^. P-Ser_46_-HPr acts as a co-factor, binding to CcpA and triggering a re-orientation that, similar to other LacI/GalR transcription factors, results in a movement of the two hinge helices of a CcpA dimer to re-position its DNA-binding domains into an orientation favorable for binding both halves of the pseudopalindroermic CRE sequence (Fig. 1B). In *Bacillus megaterium*, a key element in controlling re-orientation is the Thr_64_ switch^41^. The P-Ser_46_-HPr moiety binds to CcpA making contact with Arg_303_ to initiate a “chain-reaction” that displaces Thr_306_, breaking a hydrogen bond with Tyr_94_ that forces the repositioning of Thr_64,_ reorienting the hinge helices to convert CcpA into its high-affinity DNA-binding conformation (Fig. 1B). The key to this movement is displacement of Thr_306_, which can be mimicked by substitution with a bulky Trp residue to mimic P-Ser_46_-HPr binding, causing constitutive activation of the Thr_64_ switch^41^. To test the hypothesis that substitution with a bulky residue is sufficient to trigger the Thr_64_ switch, the key *S. pyogenes* residue (Thr_307_) was replaced through directed mutagenesis to a different bulky residue (Tyr) to create the mutant CcpA^T307Y^ that is predicted to be locked into its high-affinity DNA binding conformation and should bind to CRE sites irrespective of P-Ser_46_-HPr (Fig. 1C).

### T307Y, ΔCcpA and WT have similar growth characteristics

**Figure 2:**
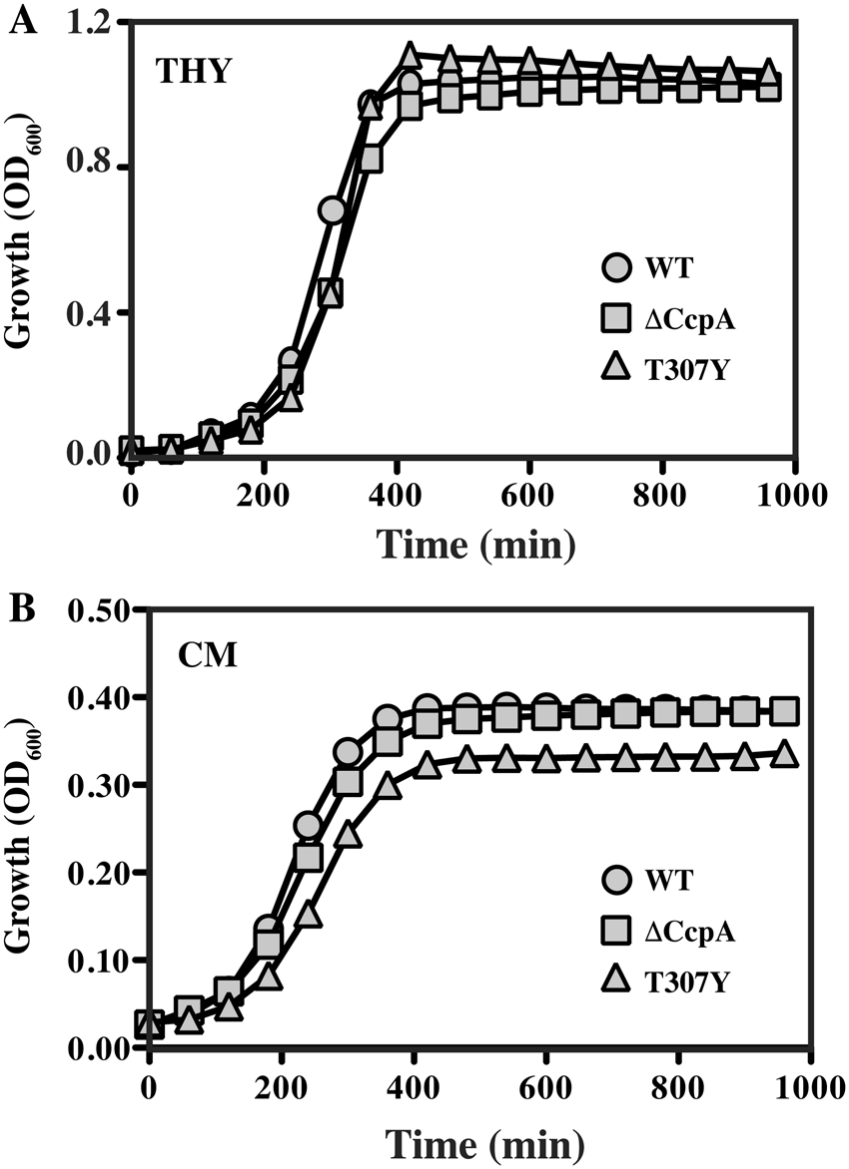
CcpA mutants lack any major growth defect *in vitro*. Growth of a mutant with an in-frame deletion of CcpA (ΔCcpA) and a mutant where resident CcpA was replaced by locked-on CcpA^T307Y^ (T307Y) were compared to WT in: (**A**) Todd-Hewitt Yeast Extract medium (THY), a glucose-rich medium; and (**B**) C medium (CM), a glucose-poor medium. Growth curves shown are from a single experiment, representative of 3 independent experiments.

To test whether manipulation of CcpA activity resulted in altered growth, a CcpA null mutant (ΔCcpA) and the CcpA^T307Y^ mutant (T307Y) were compared to wild type (WT) in several media. These included well-characterized examples of both glucose-rich and carbohydrate-limited media (THY and C-medium, respectively). For the glucose-rich medium, both CcpA mutants had growth characteristics essentially indistinguishable from WT, including length of lag phase, time of entry into stationary phase, growth rate and final growth yield (THY, Fig. 2A). Similarly, in C-medium, which transcriptomic studies indicate mimics conditions encountered in the late stages of infection in the murine subcutaneous ulcer model^21^, both CcpA mutants had growth characteristics comparable to WT, although T307Y had a slight reduction in both growth rate and final growth yield (CM, Fig. 2B).

### CcpA^T307Y^ is constitutively active in the absence of a glucose signal

**Figure 3:**
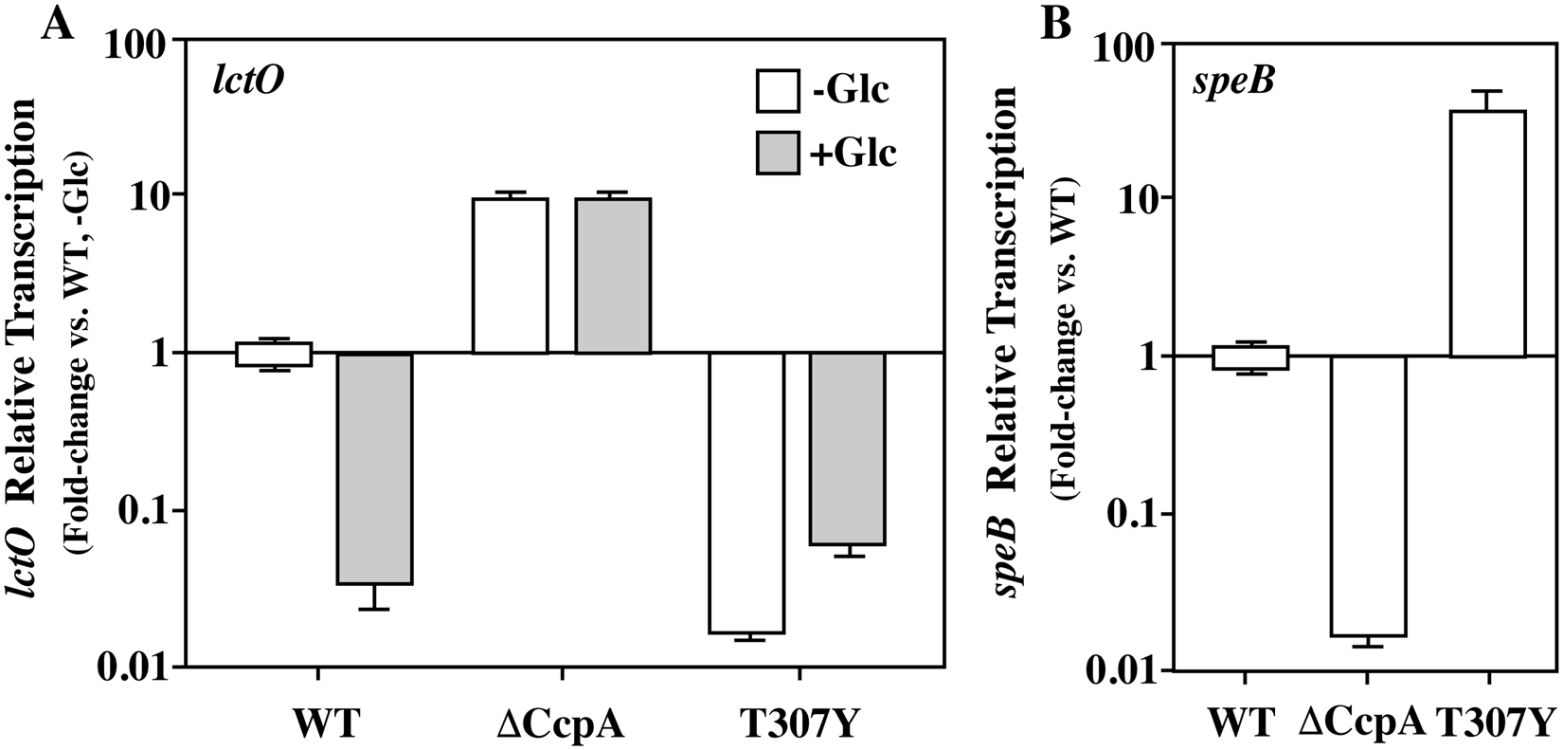
Expression of two CcpA signature genes in the locked-on mutant. Real-time RT-PCR was used to compare transcript abundance of two well-characterized CcpA-regulated genes: negatively regulated *lctO*, encoding lactate oxidase (LctO) and positively regulated *speB*, encoding the SpeB cysteine protease (SpeB), between CcpA null (ΔCcpA) and CcpA locked-on (T307Y) mutants vs. WT. Strains were cultured to late log phase in C medium, and where indicated, media either lacked (−) or included (+) supplementation with glucose (Glc). Data presented represent the mean and standard error of the mean derived from 3 independent experiments.

To test the functionality of the CcpA^T307Y^ mutant (T307Y), real time RT-PCR was performed on two genes considered biomarkers of CcpA activation and repression for *S. pyogenes*. The best characterized example of the latter is *lctO*, encoding the enzyme lactate oxidase^5^, while the former includes *speB*, the gene for the streptococcal secreted cysteine protease^4,5^. For this analysis WT, ΔCcpA, and T307Y were grown in unmodified C-medium or C-medium supplemented with 0.2% glucose. Transcript levels were measured from cells grown to late exponential phase and normalized to WT in unmodified C medium (Fig. 3). This analysis revealed that for the WT strain, the addition of glucose resulted in a greater than 10-fold inhibition of *lctO* transcription as compared to the absence of glucose (Fig. 3A). As expected, in ΔCcpA, *lctO* transcription was constitutively derepressed, as expression was insensitive to the presence or absence of glucose and was approximately 10-fold higher than WT under all conditions tested (Fig. 3A). In contrast, T307Y displayed constitutively low *lctO* transcript abundance (>10-fold decreased vs. WT, Fig. 3A), demonstrating that its ability to repress is insensitive to the presence of a glucose signal (Fig. 3A).

For examination of the positively regulated *speB*, strains were grown in C medium and transcript abundance measured at the onset of stationary phase, when *speB* is maximally expressed (Fig. 3B). Consistent with prior studies, ΔCcpA displayed reduced levels of the *speB* transcript as compared to WT (Fig. 3B). Consistent with its positive role in *speB* expression, T307Y exhibited an approximately 50-fold increase in *speB* transcript compared to WT (Fig. 3B). Taken together, these data indicate that the T307Y mutant is constitutively active in its DNA-binding conformation, and depending on the target gene, it induces either hyper-repression or hyper-activation.

### T307Y has altered virulence in a murine soft tissue infection

**Figure 4:**
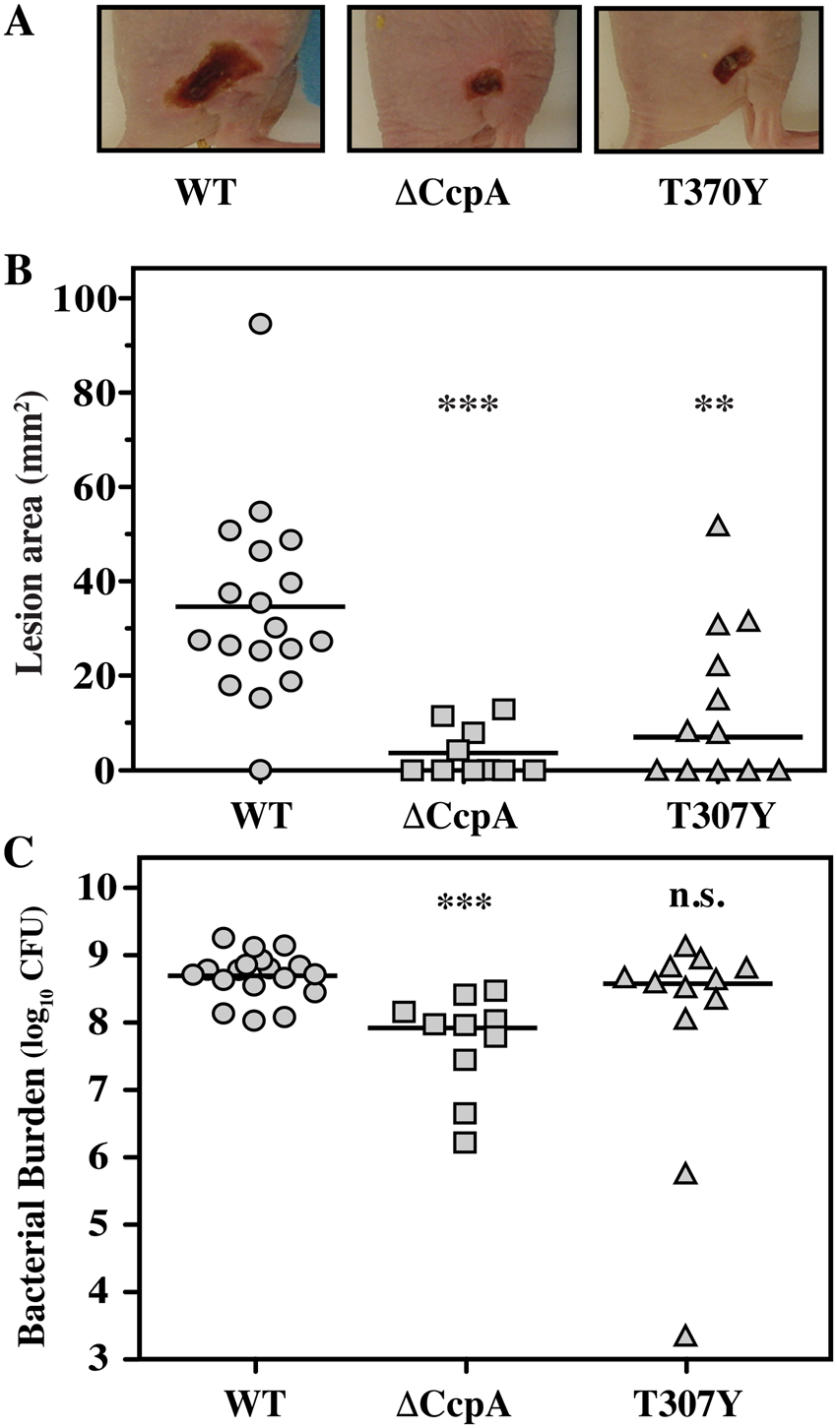
CcpA^T307Y^ mutants display uncoupled growth/damage balance in soft tissue infection. Hairless SKH1 mice were challenged subcutaneously with ΔCcpA, T307Y and WT and examined at the time of maximum ulcer formation for WT (Day 3 post-infection). Assessed were: (**A**) area of the resulting ulcer, visualized in representative mice, (**B**) ulcer areas quantitated for all infected mice and (**C**) total CFUs recovered from all infected mice. Data shown are pooled from 2 independent experiments and each symbol represents an individual mouse. Horizontal bars represent median values.

The loss of CcpA is associated with attenuation in a murine soft tissue infection model^5^. Therefore, it was of interest to determine the effects of constitutive CcpA DNA-binding activity on virulence in this model. Consistent with prior analyses, ΔCcpA was significantly attenuated for virulence vs. WT as assessed by the ability to form a lesion in the murine subcutaneous tissue (Fig. 4B). Additionally, at day 3 post-infection, the time of maximal lesion formation, the lesion area of the ∆CppA mutant was significantly reduced compared to WT (Fig. 4A,B). Similarly, T307Y was attenuated vs. WT on the basis of the ability to form a lesion (Fig. 4A,B). Based on these criteria, there was no significant difference between ΔCcpA and T307Y in their virulence properties (Fig. 4A,B). However, analyses of bacterial burdens revealed that the underlying mechanism of attenuation for ΔCcpA and T307Y was fundamentally different. Attenuation of ΔCcpA was associated with a significantly reduced bacterial burden relative to WT at Day 3 (Fig. 4C). In contrast, while T307Y was attenuated on the basis of tissue damage, this did not correlate with tissue burden, as the burden of T307Y was not significantly different from WT (Fig. 4C). Taken together, these data indicate that while ΔCcpA does not form lesions as well as wild-type, that T307Y has altered virulence, with WT levels of tissue burden coincident with an attenuated level of tissue damage.

### T307Y and ΔCcpA have opposing effects on asymptomatic mucosal colonization

**Figure 5:**
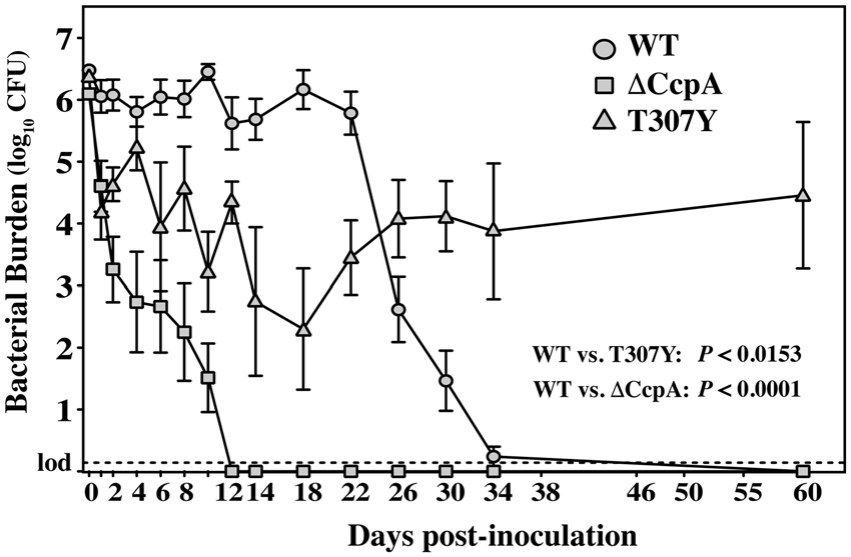
CcpA mutants display distinct patterns of carriage in an asymptomatic mucosal infection model. Estrogenized C57Bl/6J mice were vaginally inoculated on Day 0 with 1 × 10^6^ CFU of streptomycin-resistant derivatives of WT, ΔCcpA and T307Y. At the indicated time points, PBS washes of the vaginal vault were collected and were processed for determination of recoverable CFUs. At each time point shown, the data presented represent the mean and standard error of the mean derived from at least two independent experiments comprised of 5 mice per group. Differences between groups were tested for significance using a repeated measures statistic, with the calculated values for *P* indicated in the Figure.

In addition to causing inflammatory infections of the skin, *S. pyogenes* can asymptomatically colonize mucosal tissue^24^. In a model of asymptomatic carriage in the murine vaginal mucosa it was shown that a CcpA null mutant was attenuated^25^, demonstrating that CcpA regulation plays an essential role in adaptation of the transcriptome to promote long-term mucosal colonization by *S. pyogenes*. The next question to address is how locked-on CcpA may alter the characteristics of mucosal colonization in this model. Streptomycin-resistant derivatives of WT, ΔCcpA or T307Y (Table 1) were vaginally inoculated into pre-estrogenized C57BL/6J mice and colonization monitored by determination of viable streptomycin-resistant CFU recovered from vaginal washes collected over the course of 60 days. As previously seen^25^, WT maintained a high-level of colonization through day 22, after which there was a rapid drop in CFU to undetectable levels by day 34 (Fig. 5). Also consistent with prior reports^25^, ΔCcpA displayed an immediate drop in CFU, leading to complete clearance by day 12 (Fig. 5). In contrast, T307Y had a phenotype distinct from either WT or ΔCcpA. For the locked-on mutant, CFU declined gradually by several logs over the first 14 days, while overall maintaining higher numbers than ΔCcpA but lower than WT. A unique characteristic of T307Y is that over the course of the next few days, its numbers rebounded and it displayed extended carriage, with a high tissue burden (~10^5^ CFU) detected at the termination of the analysis (day 60), long after the time point at which the WT was cleared (34 days, Fig. 5). These data show that while ΔCcpA and T307Y had colonization profiles that differed significantly from WT (Fig. 5), this was due to fundamentally different mechanisms, as ΔCcpA’s ability to colonize was attenuated, while T307Y’s was enhanced.

**Table 1:** Strains used in this study.

Strain	Description (Name^a^)	Reference
HSC5	Wild type (WT)	^58^
CKB207	In-frame deletion of *ccpA* (ΔCcpA)	^4^
EP103	Allelic replacement of *ccpA* with *ccpA*^T^^307Y^ (T307Y)	This work
HSC12	Streptomycin-resistant derivative of HSC5	^25^
MEW41	HSC12 with an in-frame deletion of *ccpA*	^25^
EP109	Streptomycin-resistant derivative of EP103	This work

^a^All strains are derived from HSC5. Name refers to designation used in text and figures.

## Discussion

In this study, comparison of WT *S. pyogenes* with mutants lacking CcpA or expressing CcpA locked into its high-affinity DNA-binding conformation shows that CcpA regulates the growth/damage relationship. In general, the loss of CcpA was associated with a diminished ability to grow in tissue, leading to less damage and early clearance. In contrast, constitutive DNA-binding activity uncoupled the growth/damage relationship, with higher levels of growth attendant with reduced damage and/or extended carriage. These data provide insight into how CCR may impact pathogenesis at different tissues and how the topology of the *S. pyogenes* virulence network has evolved.

Possessing just a single alternative sigma factor whose only known role is in the regulation of competence gene expression^42^, most of the *S. pyogenes* transcriptome is under the control of a number of two-component systems and “stand-alone” response regulators (reviewed by^20,43^). Like CcpA, many of these control gene expression in response to various carbon-based cues^19,44–49^. However, how these multiple carbon-based inputs interact to influence the overall characteristics of the virulence transcriptome and their hierarchical relationships is not well understood. In this latter regard, studies in model organisms have shown that CCR plays a dominant regulatory role to promote optimal fitness in response to changing nutritional landscapes. CCR accomplishes this by adjusting growth rates in response to available carbon sources according to the “growth law” whereby the expression of carbon catabolic genes declines linearly as growth rates increase^50,51^. This adjustment optimizes the allocation of carbon between catabolic and biosynthetic pathways in order achieve an optimal rate of growth for a given set of nutritional inputs. A simple extrapolation of the growth law to pathogenesis would predict that CCR would function similarly to couple virulence gene expression to changes in growth rates.

However, CCR likely plays a more complicated role in *S. pyogenes* pathogenesis than can be explained by the growth law. For example, growth rates did not differ between CcpA mutants and WT when examined between carbohydrate-rich vs. -poor media *in vitro*. When examined *in vivo*, it was growth yield that was the most prominent difference between the pathogenic profiles of the CcpA mutants. This suggests that rather than adjusting growth rate, CCR functions to couple virulence factor expression to the hierarchical utilization of carbon source. In this latter model, the expression of defined subsets of the virulence transcriptome is linked to the presence or absence of certain carbon sources. The net effect is the temporal regulation of virulence gene expression with respect to growth phase. This model is consistent with the prior observation that disruption of CcpA function alters the temporal pattern of gene expression during infection^4,5^.

A hierarchical model can also explain why the T307Y mutant has an uncoupled growth/damage relationship, such that damage is not a linear function of growth yield. This model predicts that the early-phase genes are more focused on inhibition/avoidance of host immunity to promote growth during infection, while the late-phase genes promote inflammation and damage. Since CcpA^T307Y^ is locked into the conformation that it most likely adopts during the early stages of infection, this predicts that this mutant does not express these late-phase damage-producing genes. As a consequence, growth achieves yields equivalent to WT, but in the absence of WT levels of damage. This model also provides new perspectives on understanding the role of individual virulence factors in promoting growth/damage balance. For example, the SpeB protease has multiple proinflammatory activities suggesting it contributes to tissue damage (reviewed^52^), which is consistent with the observation that strains causing severe infection vs. those associated with the carrier state typically express high vs. low levels of SpeB, respectively (reviewed^53^). However, the T307Y mutant expresses SpeB at levels higher than WT, indicating that SpeB by itself is not sufficient to produce tissue damage in the models examined here. This raises questions as to what other virulence factors may contribute to tissue damage.

A hierarchical model that modulates the growth/damage relationship on a temporal basis is also consistent with the fact that most *S. pyogenes* infections are self-limiting, a feature reproduced by the WT strain in the two animal models examined here (reviewed^24^). Selection for self-limitation may explain the observation that while the T307Y mutant matches or exceeds WT for fitness in these models, that selection in nature cannot be described solely on the basis of optimizing fitness over a single round of infection. Other traits, including optimization of transmission between hosts, may also drive selection. Transmission is a selectable trait that can optimize pathogen fitness on a population scale^54^ and stimulation of an inflammatory response that concurrently promotes self-limitation can be adaptive in cases where it optimizes transmission to new hosts, as has been described for some respiratory pathogens^55^ and salmonellae^56^. This suggests that a CCR-based hierarchical model has been selected as a trade-off, to balance fitness in a single round of infection vs. transmission rates to optimize fitness on a population, rather than an individual, scale. However, it is well-recognized that different *S. pyogene*s strains vary significantly in virulence properties with respect to causing self-limiting vs. invasive diseases in murine models. In contrast to the strain analyzed in the present study, deletion of CcpA in a strain that is invasive in the murine ulcer model, rather than leading to attenuation, results in a hyper-virulent invasive phenotype^6^. Although there are contrasting phenotypes, a feature in common is that CcpA is regulating the growth/damage balance. Further analysis will be required to understand how strain-specific differences in the CcpA growth/damage regulatory network contribute to self-limiting vs invasive disease. The current study focused on a relatively limited subset of known CcpA-regulated virulence factors. A more comprehensive analysis of network alterations in CcpA-null and T307Y constitutive mutants between self-limiting and invasive strains may reveal that nuanced strain-specific differences in CcpA regulation may have a significant influence in altering the growth/balance damage. A precedent is the complexity of CcpA regulatory modes in *Bacillus subtilis*, where subtle changes in CcpA function can have wide-ranging effects on expression of the transcriptome^57^.

In summary, in order to achieve maximum fitness, pathogenic bacteria are challenged to balance growth with tissue damage, requiring precise coordination of expression of metabolic genes with expression of genes encoding virulence factors. This study has shown that CcpA plays a critical role in regulation of the growth/damage relationship for *S. pyogenes*. Further analysis of CcpA will provide insight into why *S. pyogenes* infection can have dramatically different degrees of damage at different tissues and will contribute to a comprehensive understanding of transcriptome dynamics during infection.

## Materials and Methods

### Bacterial strains and growth conditions

Standard molecular cloning techniques utilized the *Escherichia coli* strain TOP10 (Invitrogen), cultured in Luria-Bertani medium at 37 °C. *Streptococcus pyogenes* strain HSC5^58^, and its mutant derivatives (Table 1), were grown in Todd-Hewitt medium with 0.2% yeast extract (Difco) (THYB) or C medium, adjusted to pH 7.5 as described previously^59^. Routine culture conditions utilized sealed culture tubes incubated at 37 °C under static conditions. For culture of streptococci on solid medium, routine media were supplemented with Bacto agar (Difco) at a final concentration of 1.4% and were cultured in sealed jars under anaerobic conditions produced by a commercial gas generator (GasPak, catalogue no. 70304, BBL). For experiments utilizing glucose supplementation, a filter-sterilized 20% (w/v) stock solution was used to add glucose (Sigma) to a final concentration of 0.2%. All media used were sterilized in an autoclave prior to supplementation. When appropriate, cultures were supplemented with erythromycin (1 μg/mL, final concentration) or streptomycin (1000 μg/mL).

### Construction of mutants

All references to genomic loci are based on the genome of *S. pyogenes* HSC5^58^. A mutant with an in-frame deletion of CcpA (L897_02310) in the wild type HSC5 or the streptomycin-resistant derivative strain HSC12 was described previously^4,25^. The “locked-on” CcpA allele (CcpA^T307Y^), was generated using the Quikchange XL II mutagenesis kit (Agilent Technologies) and the PCR primers CcpA T307Y-F (GTGCTGTTAG CATGCGGATG TTGTATAAAA TCATGAACAA AAGAAGAGT) and CcpA T307Y-R (ACTCTTCTTT GTTCATGATT TTATACAACA TCCGCATGCT AACAGCAC). The modified allele was used to replace the resident CcpA allele using the allelic replacement vector pGCP213^60^ as described^61^. The streptomycin-resistant CcpA^T307Y^ strain was generated using the method described previously^25^. All molecular constructs and the chromosomal structures of all mutants were verified by DNA sequencing (Genewiz, South Plainfield, NJ) of PCR products generated using oligonucleotide primers (IDT, Coralville, IA) of the appropriate sequences.

### Isolation of RNA and transcript analysis

Transcript abundance of selected genes was analyzed as previously described^62,63^. Briefly, overnight cultures were diluted 1:25 into fresh C medium supplemented as described (see text) and harvested at mid-log phase (OD_600_ = 0.2). Total RNA was isolated using Qiagen RNeasy Mini kit per the manufacturer’s protocol. RNA was subjected to reverse-transcription (RT) PCR using iScript (Bio-Rad) per the manufacturer’s protocol. RT-PCR analysis of the resulting cDNA samples was performed using iQ SYBR Green Supermix (Bio-Rad). Primers for RT-PCR analysis of *lctO* and *speB* were reported previously^4^. Relative transcript abundance was determined using the ΔΔC_t_ method with comparison to the *recA* transcript and are presented in comparison to WT. Data shown are the means and the standard error derived from triplicate determinations of at least two biological samples prepared from independent experiments.

### Infection of mice

Infection of murine subcutaneous tissue was conducted as described previously^64,65^. Briefly, 5-to-6-week-old female SKH1 hairless mice (Charles River Labs) were injected subcutaneously with approximately 10^7^ CFU of the *S. pyogenes* strains indicated in the text. Following infection, the resulting ulcers formed were monitored over a period of several days by 1) digital photography to measure lesion areas as previously described, and 2) homogenization of excised tissue followed by plating to determine numbers of recoverable CFUs^64^. Data presented are pooled from at least two independent experiments with at least 10 mice per experimental group. The ability of strains to maintain asymptomatic colonization of the murine vaginal mucosa was measured in C57BL/6 mice, as previously described^25^. Colonization was assessed at selected time points over the course of 60 days by monitoring recoverable CFUs in a 50 μL wash of the vaginal vault. Data presented were collected from a single infection of 5–10 mice per streptococcal strain.

### Ethics statement

This study was carried out in accordance with the Public Health Service Policy on Humane Care and Use of Laboratory Animals and AAALAC accreditation guidelines. The protocols were approved by Washington University in St. Louis’ Animal Studies Committee (Animal Welfare Assurance number A-3381-01, and protocol numbers 20160070 and 201700270).

### Statistical analyses

For *in vitro* assays, differences between mean values were tested for significance using a two-tailed paired *t* test. Differences between WT and mutant strains in mouse lesion size and CFUs were tested for significance using the Mann-Whitney test. Differences in ability to form ulcers between strains were tested for significance using a Chi-square test. Differences in the duration of *S. pyogenes* vaginal carriage were tested for significance using a repeated measures statistic, as described^25^. Test statistics were calculated using the InStat module of GraphPad (version 3.06, GraphPad Software, La Jolla, CA). For all tests, the null hypothesis was rejected for *P* > 0.05. In Figures, *^,^** and *** indicates *P* < 0.01, <0.05 and <0.001, respectively.

## Data Availability

All data generated or analyzed during this study are included in this published article.
